# Revolutionizing the food industry: The transformative power of artificial intelligence-a review

**DOI:** 10.1016/j.fochx.2024.101867

**Published:** 2024-10-01

**Authors:** Vilhouphrenuo Zatsu, Angel Elizabeth Shine, Joel M. Tharakan, Dayanand Peter, Thottiam Vasudevan Ranganathan, Saqer S. Alotaibi, Robert Mugabi, Abdullatif Bin Muhsinah, Muhammad Waseem, Gulzar Ahmad Nayik

**Affiliations:** a Division of Food Processing Technology, Karunya Institute of Technology and Sciences, Karunya Nagar, Coimbatore 641114, Tamil Nadu, India; b Department of Biotechnology, College of Science, Taif University, P.O. Box 11099, Taif 21944, Saudi Arabia; c Department of Food Technology and Nutrition, Makerere University, Kampala, Uganda; d Department of Pharmacognosy, College of Pharmacy, King Khalid University, Abha 61441, Saudi Arabia; e Department of Food Science & Technology, FA & E, The Islamia University of Bahawalpur, Pakistan; f Marwadi University Research Centre, Department of Microbiology, Marwadi University, Rajkot 360003, Gujarat, India

**Keywords:** Artificial intelligence, Food sector, Fuzzy logic, Machine learning, Sensory analysis, Food safety, Food security

## Abstract

Artificial Intelligence (AI) is revolutionizing the food industry by optimizing processes, improving food quality and safety, and fostering innovation. This review examines AI's applications in food science, including supply chain management, production, sensory science, and personalized nutrition. It discusses techniques like knowledge-based expert systems, fuzzy logic, artificial neural networks, and machine learning, highlighting their roles in predictive maintenance, quality control, product development, and waste management. The integration of AI with sophisticated sensors enhances real-time monitoring and decision-making in food safety and packaging. However, challenges such as ethical concerns, data security, transparency, and high costs persist. AI is poised to advance sustainability by optimizing resource use, enhance food security through predictive analytics of crop yields, and drive innovation in personalized nutrition and supply chain automation, ensuring tailored products and efficient delivery. This paper underscores AI's transformative potential in the food industry while addressing the obstacles to its widespread adoption.

## Highlights

• AI enhances food quality, safety, and supply chain efficiency.
• AI-driven precision agriculture boosts sustainability and yields.
• Addressing data privacy, transparency, and integration costs is key for wider AI adoption.
• AI innovations will revolutionize food industry sustainability and efficiency.

## 1 Introduction

Artificial Intelligence (AI) has emerged as a revolutionary force that is redefining numerous industries in the ever-changing landscape of modern technology. Fundamentally, AI is a derivative of machine learning that has the capability to carry out operations that usually call for human intelligence. AI has already become a part of our everyday lives, helping us with anything from learning and problem-solving to interpreting natural language. AI also has the potential to improve efficiency, promote innovation and improve decision-making by processing huge amounts of data at previously unattainable speeds. Though previously deemed unthinkable, AI integration is accelerating at a breakneck pace in various industries including healthcare, banking, manufacturing, and entertainment. The fusion of technology and food is one of the many fields that benefits greatly from AI's capabilities. AI has been in the forefront of transformation in the food industry through process optimization, improvement of overall quality and safety standards and the creation of innovative culinary products (Jadhav et al., 2024). With a steep increase in the world's food consumption rates, the use of AI algorithms in food processing from farm to table promises both sustainability and better efficiency. AI has been deployed to improve supply chain management, customer experience, operational efficiency, and vehicle activity reduction thereby increasing the reach and service to customers at a low cost with the ultimate goal of achieving optimal business results (Helo & Hao, 2022a).

**Fig. 1:**
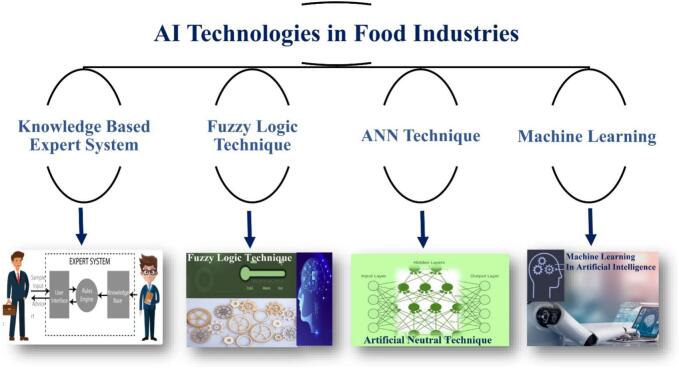
Some important AI technologies adopted by various food industries.

AI tools currently used for prediction in the food industry include the use of techniques such as gastrointestinal unified theoretical framework, partial least square, in silico models, empirical models, sparse regression, successive projections algorithms, and competitive adaptive reweighted sampling. This study is however limited to the broad use of noteworthy AI tools in the food industry. Fig. 1 illustrates several key AI technologies that have been adopted in the food industry.

### 1.1 Knowledge-based expert system

A computer software which makes use of data, information and knowledge from various sources to tackle challenging issues can be described as a knowledge based expert system. In many industries, knowledge based expert system are used in the pivotal role of collecting data and providing solution which is mimicking of the human experts' decision-making abilities. Primitive though it may be, it is regarded as one of the earliest successful AI models. This however revolves on employing specialist to assist the knowledge based expert system to resolve complex problems within a certain field. Human expert knowledge serves as the key foundation to this system. Knowledge-based Expert Systems (KBES) in the food industry are AI tools developed to replicate the decision-making capabilities of human experts by utilizing a well-organized knowledge base and inference techniques. KBES can help uphold and enhance food quality by offering expert guidance on quality control procedures. For instance, a KBES can suggest ideal storage conditions for various food products, considering their unique characteristics like temperature and humidity needs. Additionally, it can detect quality issues by analyzing production data and recommending corrective measures. KBES can be utilized to ensure adherence to safety regulations and standards. By incorporating knowledge of food safety protocols, hazard analysis, and critical control points (HACCP), these systems offer guidance on optimal practices for food handling and processing. Nestlé has utilized expert systems in product development and quality control, drawing on a knowledge base of ingredient interactions and quality standards to refine and optimize formulations.

### 1.2 Fuzzy Logic Technique (FLT)

FLT is widely used in the industry because of its ease of use and quick and precise problem-solving capabilities. By controlling human reasoning in linguistic terms, FLT has been used in the food business for food modeling, control, and classification as well as for solving food-related issues(Mavani et al., 2022). FLT could analyze factors like temperature fluctuations during transport, humidity, and ethylene levels to predict the remaining shelf life of fruits and vegetables more accurately than traditional methods. FLT can be used in sensory evaluations of bread or cakes to assess texture, flavor, and appearance, integrating subjective feedback from panels into quantifiable data for product development. Fuzzy analysis of the sensory properties of food products at various stages of unit operations can be utilized to optimize processing steps based on the desired product characteristics. In a study by Kumar, Sharma, Singh, & Singh (2015), fuzzy modeling was employed to optimize process parameters, such as soaking time, cooking time, frying temperature, and raw material properties (specifically slice thickness) in the production of taro chips. The results indicated that the experimental outcomes under optimal conditions aligned closely with the predicted values in terms of quality assessments. A sensory evaluation of four commercially available jam samples was conducted using fuzzy logic for preference ranking. The sensory attributes assessed included color, flavor, texture, and overall appearance (Shinde & Pardeshi, 2014).

### 1.3 ANN technique

Another artificial intelligence component that is frequently used in the food business is ANN. Synaptic weights, or the connections between neurons, are what allow an artificial neural network (ANN) to learn and become knowledgeable, much like the human brain (Qian et al., 2023). Goyache et al. (2001) claims that ANN is versatile, adaptive, and relevant to a variety of issues and circumstance have also reported that, despite the requirement for modifications, ANN is capable of modeling the majority of non-linear systems and is flexible enough to adapt to new circumstances.

Artificial Neural Networks (ANNs) can be trained to evaluate fish freshness by analyzing sensory data such as color, odor, and firmness, or chemical markers like pH levels. The network predicts freshness grades based on historical data, offering automated and consistent quality control. ANN models can also be employed to analyze spectroscopic data for detecting trace pesticide residues, ensuring food safety through non-destructive sample testing.

Additionally, ANNs are used to predict optimal baking times and temperatures for various breads and cakes. By examining data on dough composition, oven temperature, and humidity, the network ensures consistent quality while reducing energy usage. For dairy products like milk or yogurt, ANNs can forecast shelf life by considering factors like microbial growth, temperature changes, and packaging materials, allowing manufacturers to manage storage and distribution more effectively. Moreover, ANNs can predict the flow behavior and texture of chocolate based on its composition, such as cocoa butter content, and processing conditions like temperature, helping to optimize production and maintain texture consistency.

### 1.4 Machine learning

Machine learning (ML) is known to be the subset of AI (Goel et al., 2023). Ordinary least square regression (OLS-R), stepwise linear regression (SL-R), principal component regression (PC-R), partial least square regression (PLS-R), support vector regression (SVM-R), boosted logistic regression (BLR) and random forest regression (RF-R) are a few of the machine learning (ML) techniques used in the food industry. Studies have indicated that the application of machine learning (ML) has aided in decision-making, reduced the cost of sensory evaluation, and improved corporate strategies to better meet the needs of users (Lu, Tansuchat, Yuizono and Huynh, 2020a, Lu, Tansuchat, Yuizono and Huynh, 2020b).

Machine learning plays a pivotal role in the food industry by enhancing various processes such as predictive maintenance, food safety, and quality control. It helps optimize supply chains by forecasting demand and managing inventories, while also aiding in product development by analyzing ingredient interactions and consumer preferences. ML is crucial for personalized nutrition, shelf-life prediction, and sensory analysis, ensuring consistency in product quality. Additionally, it assists in detecting food fraud and optimizing production processes, leading to reduced waste, improved energy efficiency, and innovation across the food sector.

### 1.5 Combination of AI with external sensors

In the food industry, artificial intelligence (AI) is frequently combined with external sensors for real-time detection. These sensors include near infrared spectroscopy (NIRS), computer vision systems (CVS), electronic nose (*E*-nose), electronic tongue (E-tongue), and machine learning (ML). This allows for real-time detection and faster, more accurate results. Over the past few years, the food sectors have demonstrated a number of ways to integrate these sensors with artificial intelligence techniques (Kumar, 2023). *E*-nose, was developed to detect flavors or odors similarly to a human nose. It is made up of a variety of electronic chemical sensors that can identify both straightforward and complex smells (Qian et al., 2023). E-nose has been utilized in gas sensing applications where it is necessary to analyze individual components or mixtures of gases or vapors. (Lu, Tansuchat, Yuizono and Huynh, 2020a, Lu, Tansuchat, Yuizono and Huynh, 2020b). Furthermore, it is crucial for maintaining product quality control in the food business. *E*-tongue, which is often referred to as “a multisensory system,” has a number of low-selective sensors that are available. The signal is processed using sophisticated mathematical techniques based on pattern recognition (PARC) and multivariate data processing (Esmaeily et al., 2024). For instance, E-tongue can be used to separate different kinds of chemical compounds in liquid phase samples. E-tongue has been used to detect the umami taste in mushrooms, detect the aging of beverage flavor, and evaluate the bitterness of liquids or dissolved substances(Vilela et al., 2019). The study by Sonwani et al. (2022) introduces a novel sensor-based system for monitoring and analyzing food spoilage. The proposed device can extend the shelf life of food items and prevent spoilage by continuously tracking food quality. It alerts users through voice commands or a display and provides notifications about the predicted time remaining before spoilage occurs. The device demonstrated an accuracy of 95 % in its predictions.

## 2 Importance and application areas of AI in food science and technology

In the same way that food is essential to human survival, its science is also very important to the world. The complementary connections of studies in the domains of food science and technology as well as the food business with artificial intelligence has proven to be one of the most exciting fields for academics, scientists, and even specialists in the industrial sphere in recent decades (Liu et al., 2023). In the current food processing cycle, artificial intelligence is involved in every step of the process, from sorting to analysis and packaging. Additionally, it is highly beneficial to look at consumer satisfaction as well as issues with the food supply chain and distribution during the consumption phase (Kutyauripo et al., 2023). Popular approaches that have been used in the food sectors include expert systems, fuzzy logic, artificial neural networks (ANN), adaptive neuro-fuzzy inference systems (ANFIS), and machine learning. The application of artificial intelligence (AI) in the food business has been growing over the past few decades due to its many benefits.

Some common area in the food industry where AI is being used are mentioned below:

### 2.1 AI in supply chain management

As old as the items themselves, the idea of a supply chain has been around for a while. The supply chain's usual objectives are to build a network among many stakeholders, fulfill client demand, and enhance responsiveness. In terms of its business structure, business tasks, and stakeholders, the supply chain network is getting more and more dispersed, diversified, and transparent these days (Seyedghorban et al., 2020). The widespread application of AI has significantly improved supply chain management. AI and its applications are often among the most valuable and fascinating areas of current research. Supply chain management is evolving into an autonomous SC with self-awareness, self-government and self-determination, and self-optimization through AI integration (Helo & Hao, 2022b). The food industry is utilizing artificial intelligence (AI) to enhance supply chains by monitoring food safety and testing products at each stage of the supply chain to guarantee adherence to industry and customer requirements. Additionally, AI facilitates the transparent and efficient tracking of produce from the farm to the customer, boosting customer confidence (Chidinma-Mary-Agbai., 2020). Amazon employs AI-driven algorithms for its Amazon Fresh service, which assess customer purchasing patterns, seasonality, and market trends. Starbucks utilizes AI in its “Deep Brew” program, which factors in weather, local events, and historical data to forecast inventory needs and staffing levels (Sydoruk, 2023). Amazon utilizes AI-driven sensors to detect potential food safety hazards, such as contamination and spoilage, by monitoring the temperature and humidity of storage facilities. Similarly, Amazon Web Services (AWS) employs AI-powered image recognition systems and machine vision technologies to inspect food products for defects. AI is enhancing recommendations for grocery stores on how much of a specific product to order, helping to reduce food waste caused by over-shipping unsold items. By analyzing historical sales data, AI provides food distributors with deeper insights into what products are selling and when, allowing for more informed purchasing decisions. A 2022 study by the World Wildlife Fund found that AI software contributed to a 14.8 % reduction in food waste per grocery store. Companies specializing in merchandising technology, such as Trax and Shelf Engine, are actively involved in this area, along with major food industry players. (Cain, 2024).

### 2.2 AI in manufacturing and production

The use of artificial intelligence (AI), data analytics, and intelligent processes in manufacturing, production, and operations presents a number of difficulties and managerial consequences. There are many different types of obstacles that can arise, such as finding the necessary skills and talents for employees, adopting these applications, and dealing with a range of productivity and performance issues. For the supported supply management tasks, there are many opportunities as well as corresponding challenges. As a result, research should support operations by promoting AI approaches for clever and intelligent operations in a variety of industrial sectors while anticipating risks and weaknesses (Ore Areche et al., 2023; Papadopoulos et al., 2022).

Research on AI food safety at the production level has mostly concentrated on ML-based descriptive and predictive tools thus far. For instance, many supervised learning models have been created to predict the presence and concentrations of foodborne pathogens in agricultural water and to look into the physiochemical, temporal, geospatial, weather, and anthropogenic risk factors that are associated with these pathogens (Toro et al., 2022).

### 2.3 AI in sensory science

The goal of artificial intelligence (AI), which is founded on methods derived from nature, is to create intelligent systems that replicate elements of human behavior, including perception, learning, reasoning, evolution, and adaptation(Vilela et al., 2019). Utilizing AI technologies has become a viable solution for the sensory sciences, offering novel ways to speed up research and a fresh approach to problem-solving. Research has demonstrated that the use of AI improved corporate strategies to meet consumer needs, lowered the cost of sensory evaluation, and streamlined decision-making (Nunes et al., 2023). The majority of artificial intelligence applications in the field of sensory science are centered around the creation of regression and classification models through the use of product composition data. These demonstrate the strong correlation between non-sensory measurements, such as physical and physiological, and sensory measurements. Nunes et al. (2023) employed a random forest (RF) model to predict the acceptance, expectation, ideal sweetness, ideal acidity, and ideal succulence of strawberries. To categorize strawberry samples as “satisfactory” or “not satisfactory,” and as “would pay more” or “would not pay more,” a new RF model was fitted with the expected parameters as input. According to the findings, the approach may be applied to strawberry quality control, allowing the creation of classes and quality standards that take customer feedback into account.

Application of artificial neural network (ANN) algorithm to predict the sensory characteristics of cheese by using NIR data has been reported by Thapa et al. (2023). Based on observations like those from NIR, the authors found that ANNs can accurately anticipate sensory reactions. Currently, relatively simple AI approaches are used in sensory and consumer studies related to food goods. For example, in many ML-based investigations, local models are developed using supervised methods to study and replicate the behavior of sensory panels; these models are rather simple in comparison to the power of ML methods. When it comes to integrating AI technologies into sensory and consumer studies, there is still generally a gap between scholarly research and industry practices. This can be lessened through safe human-robot collaboration, which can take advantage of both human and robotic advantages to offer solutions that will help the food sector in general and customers in particular.

Tastewise is a food intelligence firm that leverages generative AI to assist both emerging startups and established major players in the food and beverage sector. Their services help clients stay ahead of trends, validate ideas, and foster innovation. Tastewise uses AI to analyze social media, recipe blogs, and reviews, helping food companies grasp consumer preferences and trends. It identifies popular ingredients and flavors, enabling companies to adjust their products to meet market demands and boost success.

### 2.4 AI in new product development

According to Soltani-Fesaghandis and Pooya (2018) and others, the introduction of new goods is thought to be the most important factor in a company's survival or success. Additionally, not exempt from this, are the food businesses. Similar to other businesses, there is a quick shift in the preferences of clients and the way they use new technologies in this sector (Kwong et al., 2016). The dynamic conditions that currently exist are pressuring food production enterprises to consistently innovate new goods. As a result, significant efforts have been made over the past ten years to pinpoint the variables that influence the effectiveness of new product development initiatives (Hassoun et al., 2023). The research indicates that a significant portion of the food industry's prior efforts in the creation of new products have been lost due to product failure. According to reports, new food products fail 70–80 % of the time (Dijksterhuis, 2016). Systems with artificial intelligence can assist managers in making decisions and choosing the best course of action at any point throughout the creation of new products. Managers attempt to create new products in numerous new product development initiatives in the food sector, irrespective of the company's capacity or capabilities, just because of market forces. This could therefore result in the failure of the business and the loss of several resources (Kwong et al., 2016). AI and machine learning are being increasingly adopted by global food companies for product innovation. For example, Mondelez International uses AI to accelerate the development of new products and flavors. The company first analyzes historical data to leverage existing knowledge, then employs an AI-augmented product development tool to reach optimal solutions more quickly. Mondelez plans to further integrate AI into its new product development processes, and while the technology is currently applied in a few specific product areas, its use is expanding across various categories. Similarly, Nestlé has developed an AI module for recipe development that operates under multiple constraints. Additionally, Belgium-based Foodpairing assists food manufacturers in creating new products using AI (Creasey, 2022)

### 2.5 AI in food quality and food safety operations

Food now holds international trade status, leading to numerous quality and safety concerns. Various international quality and safety standards have existed since the early days of basic quality laws several decades ago. Traditionally, assessments of food safety and quality were labor-intensive and time-consuming. However, recent technological advancements have made monitoring these issues at different stages of the food value chain easier and faster (Cannas et al., 2023). Product creation uses AI and ML extensively to ascertain user preferences. It is essential that these technologies are extensively implemented throughout processes to monitor and improve food quality and safety, as the food processing sector is expected to develop dramatically worldwide in the years to come. Food safety is crucial to human health and survival, and it merits the use of more sophisticated technology to shield businesses from harm to their reputations and customers from foodborne illnesses (Liu et al., 2023). The food sector is already greatly impacted by artificial intelligence (AI) and big data, which are seen as the fourth industrial revolution. These technologies increase food production, quality, and nutrition while decreasing resource use and waste (Sohail et al., 2018). Moreover, recent research has investigated AI-based strategies to address dietary issues that typically result in chronic illnesses like hypertension (Prakash et al., 2023).

A significant portion of the global food supply is discarded daily, often because of contamination. The IBM Supply Chain Intelligence Suite: Food Trust can help the organization to maintain food safety standards, reduce waste, and ensure regulatory compliance. IBM Food Trust employs blockchain technology combined with AI to improve food safety and traceability. This system enables real-time tracking of food products from farm to table. AI algorithms analyze data from different points in the supply chain to detect potential risks and anomalies. The system has proven highly effective in refining recall processes. For example, if a product is contaminated, IBM Food Trust can swiftly trace the problem to its origin, thereby limiting the extent of recalls and reducing consumer impact (IBM Food Trust, 2024).

### 2.6 AI in processing facilities and waste management

Food processing facilities employ environmental monitoring methods to identify contamination and confirm the efficacy of control measures. In order to simulate sampling tactics and corrective measures, recent studies have created agent-based models of Listeria contamination in food facilities(Misra et al., 2022). Model performance and decision support can be enhanced by combining machine learning with agent-based modeling (Górriz et al., 2020). Facility environmental monitoring strategies can be designed and optimized with the use of machine learning techniques. AI can also be used in the food industry to optimize and monitor sanitation and cleaning procedures. Previous research have utilized ML with various sensor technologies (e.g., electrical, optical, acoustic, and ultrasonic) to monitor fouling and cleaning of food processing equipment(Zhu et al., 2021).

Winnow Solutions is a tech company dedicated to helping businesses cut food waste and enhance efficiency. Established in 2013, Winnow's mission is to save money and benefit the environment through its AI-powered solutions. Winnow Solutions utilizes AI to address food waste in commercial kitchens. Their system features smart scales and image recognition technology to monitor and analyze food waste patterns. By offering real-time data and insights, it helps kitchens pinpoint sources of waste, adjust portion sizes, and apply waste reduction strategies. For instance, a restaurant using Winnow Solutions achieved a 30 % reduction in food waste within the first few months.

### 2.7 AI in Packaging of foods

**Fig. 2:**
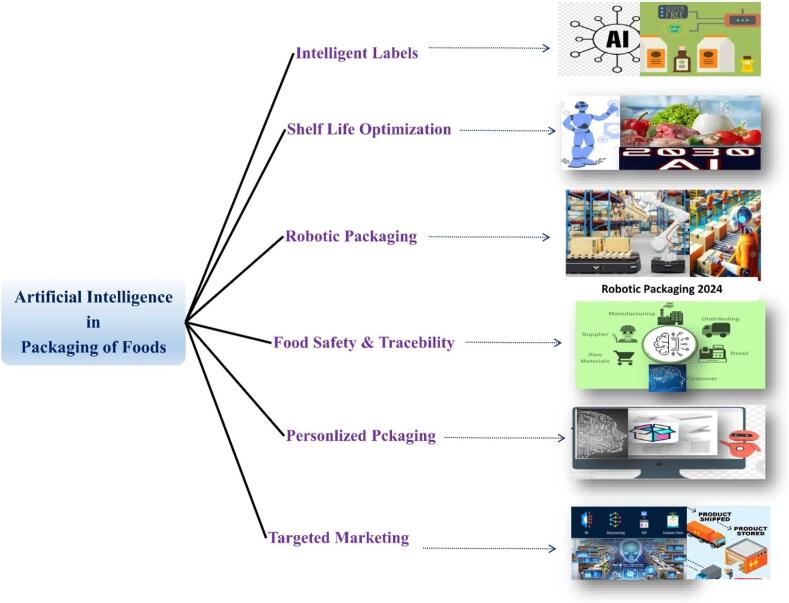
Application of artificial intelligence packaging of foods.

Artificial Intelligence (AI) plays a pivotal role in revolutionizing the packaging of foods. It optimizes various aspects of the packaging process, enhancing efficiency, safety, and sustainability (Kumar et al., 2021). AI enables smart packaging solutions, incorporating sensors and data analytics to monitor and control factors such as freshness, temperature, and shelf life. This ensures that food products remain in optimal condition throughout the supply chain. Currently, there are several advanced intelligent packaging solutions available. For instance, the University of California, Berkeley, and Stanford University have collaboratively developed intelligent packaging bags. In Germany, Kuhne has created a company that designs packaging capable of adjusting its breathability, pH, gas composition, and other parameters based on external conditions to provide real-time detection and control of food freshness. A Dutch company has developed self-regulating intelligent packaging that adapts to changes in environmental conditions. Similarly, Zhejiang University in Hangzhou, China, has produced self-regulating packaging that responds to various external factors. While these innovations have improved food freshness testing technology, addressing issues such as limitations, high costs, and slow processing speeds, there are still many areas that require further development and refinement (Li, Liu, Pu and Zhong, 2023a, Li, Liu, Pu and Zhong, 2023b). AI is transforming the food packaging industry in numerous innovative ways, as illustrated in Fig. 2.

#### 2.7.1 Intelligent labels

Dynamic labels with QR codes can give consumers real-time information about the food's origin, freshness, and nutritional content. AI can analyze data from sensors embedded in the packaging, like temperature and spoilage indicators, to alert consumers about potential issues (Li, Liu, Pu and Zhong, 2023a, Li, Liu, Pu and Zhong, 2023b)

#### 2.7.2 Shelf-life optimization

AI algorithms can analyze historical data and real-time conditions like temperature and oxygen levels to predict a product's remaining shelf life more accurately.(Sohail et al., 2018) This helps reduce food waste and optimizes distribution.

#### 2.7.3 Robotic packing

AI-powered robots can handle complex packaging tasks faster and more accurately than human workers. This reduces labor costs, increases overall efficiency, and minimizes mistakes.

#### 2.7.4 Inventory management

AI algorithms can optimize inventory levels by forecasting demand based on sales data, weather patterns, and social media trends(Khan, 2021). This reduces storage costs and ensures product availability during peak seasons.

#### 2.7.5 Enhanced sustainability and food safety

Sustainable packaging design: AI can analyze data on different materials and production methods to design eco-friendly packaging that optimizes resources and minimizes environmental impact.

#### 2.7.6 Food safety and traceability

AI algorithms can track food products throughout the supply chain, identifying contaminated batches and potential breaches in safety protocols. This helps prevent outbreaks of foodborne illnesses and builds consumer trust.

#### 2.7.7 Personalized packaging and marketing

AI can analyze consumer preferences and purchasing data to personalize packaging design and messaging based on individual demographics and interests. This creates a more engaging experience for consumers and potentially influences buying decisions.

#### 2.7.8 Targeted marketing campaigns

AI-powered analysis of consumer data can identify trends and predict market demands, allowing food companies to tailor their marketing campaigns to specific demographics and promote products more effectively (Shahare, 2022).

### 2.8 AI in resources conservation and energy reduction

Artificial intelligence (AI) is emerging as a powerful tool for resource conservation and energy reduction across various sectors in several ways:-.

#### 2.8.1 Optimizing energy consumption

*Smart grids*: AI algorithms can analyze electricity demand and weather patterns to predict energy needs and adjust generation and distribution dynamically, reducing inefficiencies and grid stress.

*Industrial energy management:* AI can monitor and analyze energy usage in factories and buildings, identifying optimal settings for equipment and operations to minimize energy waste (Dounis, 2010).

*Smart homes and buildings:* AI-powered thermostats and appliances can learn homeowner preferences and adjust energy usage for optimal comfort while minimizing consumption (Rayhan, 2023).

#### 2.8.2 Resource management and efficiency

*Precision agriculture:* Precision agriculture is an AI-driven approach specific to the agricultural stage of the food supply chain. It involves optimizing crop yields, resource use, and reducing environmental impacts, differentiating it from the food safety and quality control processes further down the supply chain. This AI drived approach can analyze soil conditions, weather data, and crop growth patterns to optimize irrigation, fertilization, and pesticide use, reducing water and resource consumption while improving yields (Helo & Hao, 2022a).

*Waste management:* AI can analyze waste streams and optimize collection routes, predict landfill capacity, and even assist in sorting and recycling processes, maximizing resource recovery and minimizing waste generation (Kutyauripo et al., 2023). According to McKinsey, AI has the potential to address these issues and unlock significant opportunities by reducing food waste by 2030. Such remarkable outcomes can be achieved by implementing additional regenerative farming practices (Filimonau et al., 2020).

*Water management:* AI can monitor water usage patterns and leakages, optimize irrigation systems, and predict water needs based on weather and agricultural data, ensuring efficient water allocation and conservation (Li, Liu, Pu and Zhong, 2023a, Li, Liu, Pu and Zhong, 2023b).

#### 2.8.3 Renewable energy integration

*Grid integration:* AI can manage the intermittent nature of renewable energy sources like solar and wind by forecasting generation, optimizing storage deployment, and balancing grid stability with renewable energy integration.

*Renewable energy site selection:* AI can analyze geographical data, wind patterns, and solar radiation to identify optimal locations for installing renewable energy infrastructure, maximizing efficiency and resource utilization (Rayhan, 2023).

### 2.9 AI in Marketing and sales of food products

Artificial intelligence (AI) is shaking up the food industry, and its impact on marketing and sales is particularly significant (Shahare, 2022).

#### 2.9.1 Personalized targeting and engagement

##### 2.9.1.1 Data-driven customer insights

AI algorithms analyze vast amounts of data from purchase history, online behavior, and social media to create detailed customer profiles. This allows companies to personalize marketing messages, product recommendations, and promotions for each individual, increasing engagement and conversion rates.

##### 2.9.1.2 Dynamic content and advertising

AI can create custom ads and marketing materials tailored to individual preferences and contexts. Imagine recipe suggestions based on dietary needs or targeted ads showcasing products a customer recently viewed.

##### 2.9.1.3 Chatbots and virtual assistants

AI-powered chatbots can answer customer questions, provide product information, and even personalize offers in real-time, enhancing customer service and brand loyalty.

##### 2.9.1.4 Demand forecasting

AI can analyze historical sales data, trends, and external factors like weather and holidays to predict future demand for specific products. This helps companies optimize inventory levels, pricing strategies, and promotional campaigns.

##### 2.9.1.5 Dynamic pricing and promotions

AI algorithms can adjust prices and promotions in real time based on demand, competitor activity, and customer behavior, maximizing revenue and profit margins.

##### 2.9.1.6 Salesforce optimization

AI can analyze sales call data and customer interactions to identify patterns and suggest optimal strategies for closing deals. It can also automate repetitive tasks, freeing up salespeople to focus on building relationships and closing deals. AI can also reduce marketing and sales costs by automating tasks and optimizing campaigns.

### 2.10 AI in QSR (Quick service restaurants) and Cafetarias

Artificial Intelligence (AI) is spearheading a technology revolution in the rapidly changing Quick Service Restaurant (QSR) market that is transforming the way industry heavyweights like Domino's, Subway, and McDonald's operate. In addition to improving client experiences, AI is radically changing how these businesses operate at their core.

#### 2.10.1 Making things faster and smoother

Faster and more effective service in QSRs is powered by AI. Artificial Intelligence (AI) guarantees that customers receive their orders precisely and swiftly by streamlining drive-through experiences and providing real-time order tracking on mobile devices. This is about streamlining and simplifying the dining experience, not about speed. Less Hassle, More Human Touch (I. Kumar et al., 2021).

#### 2.10.2 Getting orders right and managing supplies smartly

Artificial Intelligence (AI) improves order accuracy and streamlines inventory control. Using data, QSRs are able to predict client preferences, modify pricing tactics, and maintain ideal stock levels—all of which effectively reduce waste and avoidable expenses (Prakash et al., 2023). It all comes down to correctly placing orders the first time and wisely handling supplies.

#### 2.10.3 Smart staffing and learning from sales data

AI analyzes sales data to help QSRs make well-informed decisions about personnel numbers during peak hours. This strategic approach to staffing guarantees enough workers to manage peak times, enhancing both customer happiness and operational effectiveness.

#### 2.10.4 Fraud detection

A recurring issue for quick-service restaurants is several types of theft. The threat is substantial since there are potential for employees to take advantage of cash register capabilities, which might result in a 7 % loss of sales. But artificial intelligence (AI) technology is emerging as a game-changing remedy. AI becomes a vital ally for quick-service restaurants in redesigning the theft prevention strategy, protecting against financial losses and guaranteeing operational integrity (Ahamed & Vignesh, 2022).

#### 2.10.5 Employee performance enhancement

AI is capable of analyzing employee interactions and offering insightful feedback for enhancements. By rating and enhancing individual performance, this data-driven strategy promotes a competitive and healthy environment that eventually helps brands succeed. Because of this capability, AI is a vital ally in fostering employee development.

#### 2.10.6 Knowing what your customers like

Artificial Intelligence functions as a background assistant that picks up on and comprehends user preferences. With the use of this information, QSRs can recommend menu items to customers based on their previous orders, adding a customized touch that improves the entire eating experience. QSRs are entering a new era of efficiency and customer happiness as big firms like Taco Bell, Starbucks, and Chipotle embrace AI. It's more important to make useful upgrades that improve customer satisfaction and save time than to dazzle stockholders with cutting-edge technology.

### 2.11 AI in personalized nutrition

Personalized nutrition leverages individual genetic information to suggest tailored dietary habits (Pray, 2018), which could have a substantial impact on disease prevention and overall health enhancement. Incorporating AI applications in nutrition drives technological advancements that are transforming the field of dietary interventions (Detopoulou et al., 2023). AI techniques offer great potential in this data-driven age for revolutionizing our approach to understanding, monitoring, and optimizing nutritional outcomes. Kirk et al. (2022) offer a thorough overview of machine learning in nutrition research, distinguishing various machine learning methods and showcasing their applications in precision nutrition and metabolomics. Conversely, Zhu and Wang (2023) highlight the global issue of nutritional security, detailing AI applications in personalized nutrition recommendations and illustrating examples such as predicting the correlation between cooking parameters and nutritional quality. Van Erp et al. (2021) tackle the food challenge by exploring the advantages of natural language processing and AI in recipe analysis for personalized recommendations. Honda and Nishi (2021) propose a system based on purchase history data to support healthy diet choices, featuring a household nutrition analysis and food recommendation system. Additionally, Santhuja et al. (2023) present an intelligent, personalized nutrition guidance system that leverages IoT, machine learning algorithms, and image processing for real-time, customized nutrition recommendations. Maurya et al. (2019) focus on automating the classification of chronic kidney disease by providing tailored diet plans. Iheanacho and Vincent (2022) introduced a system that employs deep learning, particularly convolutional neural networks (CNN), to classify and recommend healthy food recipes tailored to local dietary habits in West Africa. The combined findings of these research papers highlight the potential of incorporating artificial intelligence techniques into nutrition research to develop personalized, context-aware solutions that enhance diet choices and promote healthy eating

## 3 Patent trend related to AI and the food industry

Patent analysis of “AI and the Food Industry” in global databases reveals extensive applications of AI, particularly in enhancing food sorting, food safety, food nutrition, food delivery, and supply chain management. The findings indicate that the leading jurisdictions for patent filings in this domain are China, the USA, South Korea, and Japan. The study also shows that the majority of patents (26 %) are related to the application of AI in cooking devices or kitchen appliances. This is followed by AI applications in health monitoring (15 %), the restaurant sector (13 %), and agriculture (10 %) (Gupta et al., 2023). The application of AI in developing various automatic cooking and kitchen appliances represents a significant advancement of this era. Notable inventions include the automatic meat floss machine (CN101218963B), smart vegetable cooking system (CN108806095), and food processing methods based on image identification (CN108303920A). Other innovations are intelligent cooking devices (CN100506128C, CN107788818A, JP2007541650A, EP1532902B1, INA201941030758), intelligent kitchen robots (CN105150221B, US9815191B2, KR20190098936A), and intelligent microwaves (US20190261459A1). There are also developments like modifying recipes to achieve specific colors for final dishes (US10332276). These inventions enhance convenience by reducing labor and saving time (Gupta et al., 2023). The growing consumer preference for easy-to-use cooking appliances suggests that the food industry should invest more in this category. Patents filed in the post-COVID-19 period highlight a rising trend in innovation within the field. Examples include:

• Smart Water Body Cleaning Hive Robot (IN202041008110)
• AI-Enabled Bot for Disposal of Food Waste (IN202041007777)
• System for Quality Check of Cooked and Packaged Food (IN202011008924)
• Wearable Body Monitor (US20200188732)
• Feeding Robot (CN111070223A)
• Meal Investigation and Evaluation Method (CN111127545A)
• Temperature Defect Detection Method in the Food Industry (CN111044177A)
• Method for Detection of Food Freshness and Food Storage Equipment (CN111209849A)

These patents reflect a notable increase in filings related to food technology and automation in recent years.

## 4 Issues and concerns of AI utilization in Food industry

**Fig. 3:**
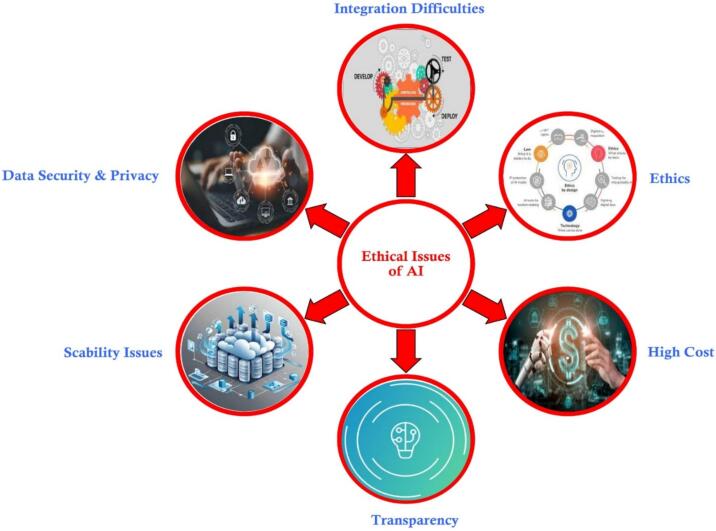
Issues and concerns of AI utilization in Food industry.

The complex business of food processing involves organizing the food or raw materials from the household, maintaining the hardware and various types of equipment, and more. Transparency, traceability, explainability, interpretability, accessibility, accountability, and responsibility are some of the specific ethical issues that apply to computational and artificial intelligence (AI) systems, even though the nature of data in the food system is far broader than the personal data protected by the General Data Protection Regulation (GDPR) of the European Union. Fig. 3 highlights some key challenges and concerns related to the implementation of AI in the food industry.

### 4.1 Data security and privacy

AI systems depend significantly on data, which may contain sensitive details about both consumers and operations. Safeguarding this data is essential, as security breaches could result in financial losses and harm to the company's reputation. The food industry needs to adopt strong cybersecurity practices to defend against data theft and comply with privacy regulations.

### 4.2 Ethics

Unfair or discriminatory results may result from AI systems unintentionally maintaining biases found in training data. It is important to guarantee the ethical use of AI in decision-making procedures, particularly in relation to recruiting, product suggestions, and customized dietary guidance. The use of AI in the food industry raises ethical questions, such as the potential for job displacement and the transparency of AI decision-making. Additionally, there may be regulatory hurdles related to the deployment of AI technologies, especially in areas like food safety and consumer protection.

### 4.3 Transparency & explainability

Because deep learning models are so complicated, they are frequently referred to as “black boxes” in the context of artificial intelligence. It can be difficult to comprehend how specific decisions are made in AI decision-making processes due to a lack of explainability and transparency, which raises questions regarding responsibility and trust.

### 4.4 Standardization and regulation

Safety, quality control, and compliance issues may arise if there are no established rules governing AI applications in the food business. To ensure responsible and ethical use, it is imperative to establish clear norms and criteria for the development and implementation of AI technology (Huberts, 2018).

### 4.5 High costs

Implementing AI solutions can be costly. The initial expenditures on technology, infrastructure, and expertise can be considerable. Moreover, continuous maintenance, updates, and training are necessary to ensure the AI systems function properly. For smaller businesses or startups, these expenses can pose a significant hurdle to adoption.

### 4.6 Integration difficulties

Incorporating AI into existing systems and workflows can be difficult. Many companies struggle to align AI technologies with their current processes and infrastructure. Achieving seamless integration demands meticulous planning, and incompatibility between new AI systems and legacy systems can obstruct the implementation process.

### 4.7 Scalability issues

Scaling AI solutions from pilot projects to full-scale implementation can be difficult. Challenges include ensuring consistent performance across different locations, adapting AI models to diverse environments, and managing the increased complexity of larger systems.

## 5 Global variations in AI adoption in the food industry

AI adoption in the food industry varies significantly across regions due to differences in infrastructure, regulations, and economic priorities. In **North America**, advanced AI infrastructure supports its use in agriculture, retail, and personalized nutrition, though concerns about data privacy and transparency remain. **Europe** focuses on sustainable and ethical AI, particularly in food safety and supply chains, but faces regulatory barriers that can slow innovation. In **Asia**, China leads with government-backed AI initiatives in food production and retail, while India leverages AI for agricultural challenges. Japan and South Korea are known for robotics in food processing, though high costs and regulations present challenges. The **Middle East** is investing in AI to improve food security through technologies like smart irrigation, while **Africa** uses AI to enhance agriculture, although limited infrastructure and regulations slow progress. **Latin America** embraces AI for agriculture and food safety, particularly in large-scale farming, but struggles with investment and infrastructure. In **Oceania**, AI-driven precision agriculture and sustainable farming practices are advancing, though geographic isolation poses challenges. Overall, while AI adoption in the food industry is growing globally, each region faces unique opportunities and challenges shaped by its regulatory, economic, and technological landscapes.

## 6 Conclusion

The integration of Artificial Intelligence into the food industry has brought about significant advancements in enhancing food production, quality control, safety, and efficiency. AI tools like knowledge-based expert systems, fuzzy logic, artificial neural networks, and machine learning have revolutionized various aspects of the food supply chain, from processing to personalized nutrition. These technologies offer substantial benefits in optimizing processes, reducing waste, and ensuring compliance with food safety standards. Additionally, the combination of AI with external sensors has enabled real-time monitoring and analysis, contributing to smarter packaging and more accurate shelf-life predictions. However, while AI presents transformative potential, its full-scale adoption remains hindered by challenges such as high implementation costs, data security concerns, lack of transparency, and the complexity of integration with existing systems. Ethical considerations, including privacy and fairness in AI decision-making, further complicate the landscape. For AI to be widely embraced in the food industry, these issues must be addressed through well-defined regulations, transparency initiatives, and efforts to make AI technology more accessible to smaller players. In conclusion, AI stands at the forefront of revolutionizing the food industry. With continued innovation, ethical deployment, and strategic integration, AI has the potential to significantly improve the efficiency, safety, and sustainability of the global food system, making it better equipped to meet the growing demands of the future. The future landscape of AI applications in the food sector will be significantly shaped by continuous research, interdisciplinary collaboration, and ethical considerations as the field continues to develop. A more robust, effective, and creative food ecosystem will surely result from the responsible adoption of this revolutionary technology.

## 7 Future scope

The rising popularity of artificial intelligence is shedding light on the food industry's future. As AI continues to evolve, its application in the food industry holds immense potential for further development. The integration of more sophisticated AI tools, such as deep learning and advanced robotics, could significantly improve food safety, quality, and efficiency across all stages of the supply chain. In the future, AI is expected to play a pivotal role in enhancing personalized nutrition by providing real-time dietary recommendations based on an individual's genetic makeup, lifestyle, and health data, thereby transforming public health strategies. The advancement of AI-driven sensory technologies, such as more precise electronic noses and tongues, will further enhance the accuracy of food quality assessments, enabling non-destructive testing of food products. Innovations in AI-powered smart packaging, which can autonomously monitor and maintain optimal conditions for food storage, will also contribute to extending shelf life and reducing food waste on a global scale. Another promising area is the use of AI in sustainable food production. AI-driven precision agriculture, combined with IoT and big data analytics, will optimize resource use, reduce environmental impact, and increase crop yields. Furthermore, AI has the potential to improve food waste management through predictive algorithms that minimize overproduction and spoilage.

However, the widespread implementation of AI in the food industry will require the development of standardized regulations, ensuring that AI systems are transparent, ethical, and safe. Future research must focus on addressing challenges such as data security, privacy, and ethical concerns surrounding AI decision-making. Collaboration between industry, government, and academia will be essential to harness the full potential of AI in transforming the global food system. In the coming years, as AI continues to mature and become more accessible, its role in revolutionizing food science and technology will expand, offering innovative solutions for enhancing food security, sustainability, and public health worldwide.

## CRediT authorship contribution statement

**Vilhouphrenuo Zatsu:** Writing – review & editing, Writing – original draft, Resources, Methodology, Investigation, Data curation, Conceptualization. **Angel Elizabeth Shine:** Writing – review & editing, Writing – original draft, Software, Methodology, Data curation, Conceptualization. **Joel M. Tharakan:** Writing – review & editing, Writing – original draft, Visualization, Validation, Formal analysis, Data curation, Conceptualization. **Dayanand Peter:** Writing – review & editing, Writing – original draft, Resources, Investigation, Data curation, Conceptualization. **Thottiam Vasudevan Ranganathan:** Writing – review & editing, Writing – original draft, Supervision, Project administration, Investigation, Formal analysis, Data curation, Conceptualization. **Saqer S. Alotaibi:** Writing – review & editing, Writing – original draft, Project administration, Data curation, Conceptualization. **Robert Mugabi:** Writing – review & editing, Software, Project administration, Funding acquisition, Formal analysis, Data curation, Conceptualization. **Abdullatif Bin Muhsinah:** Conceptualization, Formal analysis, Funding acquisition, Resources, Software, Writing – review & editing. **Muhammad Waseem:** Formal analysis, Investigation, Methodology, Software, Validation, Visualization, Writing – review & editing. **Gulzar Ahmad Nayik:** Writing – review & editing, Writing – original draft, Validation, Supervision, Software, Formal analysis, Data curation.

## Declaration of competing interest

The authors declare that they have no known competing financial interests or personal relationships that could have appeared to influence the work reported in this paper.

## Data Availability

Data will be made available on request.
